# Review of "Bird Flu: A Virus of Our Own Hatching" by Michael Greger

**DOI:** 10.1186/1743-422X-4-38

**Published:** 2007-04-30

**Authors:** Chengfeng Qin, Ede Qin

**Affiliations:** 1 State Key Laboratory of Pathogen and Biosecurity, Institute of Microbiology and Epidemiology, Beijing 100071, China

Due to my responsibility as member of advisory committee on pandemic influenza, I regard any new publication on bird flu with special enthusiasm. A book that recently caught my eye was one by Michael Greger titled **Bird Flu**: A Virus of Our Own Hatching. This book paints a global picture of how the mild bird flu in poultry developed into a devastating disaster in human and details how we prepare, survive and prevent future influenza pandemics.

The book is clearly written and highly informative. Greger's superb story-telling ability makes every page of the book interesting and fascinating for both specialist and layperson. Greger's book is logically organized into 5 sections, Storm Gathering, When Animal Viruses Attack, Pandemic Preparedness, Surviving the Pandemic, and Preventing Future Pandemics, consisting of 21 chapters.

The unifying theme of the book is that pandemics aren't born – they're made, made by ourselves. The subheading of the book leaves no doubt the conclusion that we are all going to pay the price for what we have done. An overall emphasis was laid on the consequence of modern poultry industry. Chickens and other commercial birds are raised in closed, crowded, stressful and unsanitary industrial poultry facilities, offering the bird flu virus opportunities for infection, mutation and spread. The drastic changes in poultry farming worldwide will trigger the inevitable pandemic. Fortunately, it is not so harrowing, as the author states in the introduction of book, "If changes in human behavior can cause new plagues, changes in human behavior may prevent them in the future".

Yes, we can change. In the last sections of the book, Greger carefully details how to protect ourselves in the very likely event that a bird flu pandemic begins to sweep the world and how to prevent future pandemics. Dr. Greger's simple and practical suggestions are invaluable for both nation and individual. The poultry industry should change, and "humanity must shift toward raising poultry in smaller flocks, under less stressful, less crowded, and more hygienic conditions, with outdoor access". Governments should make efforts to support scientific research on vaccines and antiviral drugs. Individuals can help to protect themselves by following Greger's advice – wash hands and preparing at home. He provides a useful check list of items to store in the event of pandemic. It should work.

Another strength that makes this book special is that Greger cites every source of information. A total of 3,172 references (more than 100 pages) are provided for further reading, mainly from scientists and governments. Numerous quotes, pretty illustrations, vivid titles and clear writing make reading really a pleasure.

In my opinion, Greger's book is the best of its genre and deserves to be read by anyone who is concerned about human and animal health. This book is a must read for government and enterprise officials who are advocating and advancing poultry industry standards. Virologists and epidemiologists will also find it useful, since the basic science of bird flu is carefully set out. I highly recommend it.

